# Threshold of the upper airway cross-section for hypopnea onset during sleep and its identification under waking condition

**DOI:** 10.1186/s12931-019-1250-4

**Published:** 2019-12-11

**Authors:** Hongyi Lin, Cunting Wang, Han Zhang, Huahui Xiong, Zheng Li, Xiaoqing Huang, Changjin Ji, Junfang Xian, Yaqi Huang

**Affiliations:** 1 School of Biomedical Engineering, Capital Medical University, 10 Xitoutiao, Youanmenwai, Beijing, 100069 China; 2 Beijing Key Laboratory of Fundamental Research on Biomechanics in Clinical Application, Capital Medical University, Beijing, China; 3 Department of Radiology, Beijing Tongren Hospital, Capital Medical University, No 1 Dongjiaominxiang Street, Beijing, 100730 China

**Keywords:** Obstructive sleep apnea, Threshold of airway caliber, Hypopnea onset, Shape change of airway

## Abstract

**Background:**

There is currently no method that can predict whether or under what condition hypopnea, even obstructive sleep apnea (OSA), will occur during sleep for individuals based on credible parameters measured under waking condition. We propose a threshold concept based on the narrowest cross-sectional area of the upper airway (CSA-UA) and aim to prove our hypothesis on the threshold of the area for hypopnea onset (TAHO), which can be used as an indicator of hypopnea onset during sleep and measured while awake.

**Methods:**

We performed magnetic resonance imaging for 20 OSA patients to observe CSA-UA changes during fluid accumulation in the neck caused by elevating their legs, and identified TAHO by capturing the sudden enlargement in CSA-UA. Correlation analyses between TAHO and the body mass index (BMI), and between the reduction in CSA-UA and the increase in the neck circumference (NC) with fluid accumulation were performed. Logistic regression analysis was performed for identifying OSA patients based on the behaviors of their CSA-UA changes during leg raising. Shape changes of airway cross-section were also investigated.

**Results:**

Four CSA-UA change patterns after fluid redistribution were identified. Six patients had similar CSA-UA variation behaviors observed in healthy subjects. From the other three change patterns involving 14 patients, a threshold value of CSA-UA 0.63 ± 0.21 cm^2^ was identified for normal breathing. Data showed a positive correlation between TAHO and BMI (r = 0.681, *p* = 0.0007), and a negative correlation between the reduction in CSA-UA and the increase in NC (r = − 0.513, *p* = 0.051) with fluid accumulation. A sigmoid function for the probability of being a OSA patient *p* = 1/[1 + exp. (4.836 + 3.850 t-8.4 h)] was obtained to effectively separate OSA patients from normal subjects. The upper airway narrowing occurred in anteroposterior, lateral, or both directions, suggesting different tendencies of upper airway collapse in patients. Three types of shape changes in the cross-section of the upper airway, which had different effects on airway resistance, were measured.

**Conclusions:**

Our findings prove TAHO hypothesis. The threshold measured while awake for normal breathing can be used clinically as the indicator of hypopnea onset during sleep, and therefore to identify OSA patients under waking condition and design effective personalized treatments for OSA patients. Both shape and size changes in the cross-section of the upper airway affect airway resistance significantly. Shape change in the cross-section of the upper airway can provide key clinical information on the collapse patterns of the upper airway for individuals.

## Introduction

As a common disorder of the respiratory system, the awareness rate of obstructive sleep apnea (OSA) and its complications in the general population is low [1, 2]. Many patients suffering from OSA have not been clinically diagnosed and treated [3–5]. The current methods for OSA diagnosis are quite limited, and mainly depends on overnight polysomnography to measure the apnea-hypopnea index (AHI). Clinicians have insufficient tools to evaluate OSA under waking condition. When designing a personalized treatment for patients, such as a surgery, one needs credible methods to predict its effectiveness before performing such a treatment. Medical imaging is one of the most effective techniques to observe the patency of the upper airway (UA). However, due to difficulties in head and neck imaging under sleep condition, and the large difference in UA patency between being awake and asleep [6, 7], currently medical imaging can only provide very limited information for OSA evaluation. There is no method that can predict whether or under what condition hypopnea, even OSA, will occur during sleep for individuals based on credible parameters measured under waking condition. If one can find such parameters or indexes that can be measured under waking condition, it will be a great help for OSA screening or the designing of personalized treatments through credible pre-evaluations on treatment outcomes.

UA is the channel to supply oxygen to the human body. To provide enough oxygen for organs and tissues, a certain air flux is needed during breathing. From the physiological and mechanical points of view, the resistance of UA strongly depends on UA geometry [8, 9]. UA resistance will increase with the airway collapse. When UA is too narrow to allow enough air passage, hypopnea will occur. For an air tunnel with varied cross-sectional area, the narrowest part contributes the most to flow resistance. Therefore, the narrowest cross-sectional area of UA (CSA-UA) is a potential parameter to characterize UA behaviors.

Although the exact threshold value of CSA-UA may vary among individuals, one rule is clear: to provide organs with sufficient oxygen, CSA-UA must not be smaller than a critical value in order to maintain normal breathing. CSA-UA is closely related to the activation of pharyngeal dilator muscles. Studies have revealed that while awake, enhanced dilator muscle activations can maintain the airway size for normal breathing. The muscle activations in OSA patients are much stronger than those for healthy people under waking condition because the passive UA is narrower in patients [10–14]. However, this ability of genioglossus at daytime will largely be lost during sleep [15, 16]. Without the help of strong muscle activations, the wall of UA cannot withstand the negative pressure during inspiration, and UA will collapse. If UA becomes too narrow, hypopnea or even apnea will occur. Based on the above observations, we propose a new concept related to CSA-UA: the threshold of the area for hypopnea onset (TAHO). For each individual, there is a personal threshold value of CSA-UA. Once CSA-UA is below TAHO due to weak dilator muscle activation during sleep, the patient becomes prone to hypopnea and even OSA.

However, it is a big challenge to measure TAHO under waking condition. We propose the following hypothesis: While awake, once CSA-UA reaches TAHO, muscles will activate in order to enlarge UA immediately to avoid the occurrence of hypopnea. Therefore, if one can gradually increase the tissue volume surrounding UA in a proper way to narrow the airway, a sudden enlargement of CSA-UA will appear at TAHO due to a rapid increase in muscle activations. The minimum value of CSA-UA observed right before the triggering of the strong muscle activation can be considered TAHO.

Our previous MRI study in healthy subjects under waking condition shows that when the legs of subjects lying supine are elevated, the fluid in the lower body will shift into the head and neck region due to gravity. This process increases the volume of the soft tissues, and therefore compresses the airway to reduce CSA-UA [17]. This natural process of compressing the airway gradually to reduce CSA-UA is easy to control and perfectly matches the requirements for CSA-UA measurements. Although there are differences in the magnitude of CSA-UA reduction among individuals due to the modulation of their different skin elasticities [18], UA is continuously narrowed under the tissue stress generated by the volume expansion of the surrounding tissues when the amount of fluid in the neck region is increased. Because the original CSA-UA is large in healthy subjects, the fluid amount increased in the neck region due to the leg raising is not large enough to reduce CSA-UA to TAHO. Therefore, CSA-UA keeps decreasing during the 8 min of leg raising in these healthy subjects [17]. However, the case will be different for OSA patients. Because the original size of UA cross-section is much smaller in patients than in healthy people [19–21], it will be easy for CSA-UA to reach TAHO during fluid accumulation in the neck region, which will provide a good opportunity for us to measure TAHO under waking condition. We may expect to observe a sudden increase in CSA-UA of patients when performing MRI with the leg raising. It means that there is an inflection point on the curve of CSA-UA versus fluid amount increment. The minimum value of CSA-UA curve is precisely the TAHO that we want to identify.

In this study, we aim to prove our hypothesis by capturing the sudden enlargement in CSA-UA of OSA patients using a convenient and non-invasive method based on MRI, and therefore to measure TAHO under waking condition at daytime. Meanwhile, we also want to classify the types of UA obstructions in patients by observing the shape changes in the cross-section of UA during airway collapse.

## Methods

### Subjects

Potential OSA candidates not previously treated for OSA were recruited. Patients with histories of cardiovascular or neurological disease were excluded. An overnight polysomnography study was performed for all potential candidates using Alice 5 Diagnostic Sleep System (Philips Respironics, Inc., USA). Patients with apnea-hypopnea index ≥15 participated in the MRI study because the changes in their airway cross-sectional area could be more obvious, and therefore, it should be easier to observe and measure TAHO. The research protocol was approved by the Ethics Committee of the Capital Medical University, Beijing, China (2013SY67). All subjects provided written informed consent prior to participation.

### Magnetic resonance imaging protocols

Using a spoiled gradient echo sequence used previously for healthy subjects [17], three-dimensional 3.0 Tesla MRI was performed (Signa HDxt, General Electric, USA) for participants while awake. The time spent on axial plane imaging from the top of nasal cavity to the laryngeal prominence was about 150 s.

Each participant lay supine on the MRI sliding bed with their head fixed in the neutral position. After a 15-min relaxing, the patient was moved into the device. Scans, starting at the end of a tidal expiration, were performed under four conditions: baseline while lying supine, 1 min and 8 min after elevating both legs by more than 40°, and then immediately after lowering the legs. The total time spent in the MRI device was about 16 min.

### Data extraction

To reduce the measurement error, instead of identifying the image with the narrowest caliber of UA in each of the four scanning stages, we use a mean value to define CSA-UA for each stage. For each participant we selected ten consecutive axial images in the narrowest part of the retropalatal airway, which cover 1.3 cm in length for each scanning stage at the same location. Therefore, 40 images were selected for each participant. We enlarged the images and outlined the boundaries of UA manually. We then extracted their coordinates to calculate CSA-UA for each scanning stages. For each participant, CSA-UA was calculated by averaging the ten corresponding cross-sectional areas for a given stage. The shapes of UA cross-section under different conditions were also collected.

### Data analysis

A power analysis was performed to estimate the required patient size for this study. Measured values were expressed as means ± standard deviations. A curve of the change in CSA-UA with 4 scanning status was plotted for each patient. The minimum value of CSA-UA curve during leg raising was considered as TAHO. Correlation analyses between TAHO and the body mass index (BMI), and between the reduction in CSA-UA and the increase in the neck circumference (NC) with fluid shift were performed to observe their relationships. A logistic regression analysis was performed for identifying OSA patients based on the behaviors of their CSA-UA changes during leg raising. The logistic regression model was established based on the data of the patients as well as of normal subjects from our previous fluid shift study [17]. For normal subjects and any OSA patient who could not reach the CSA-UA threshold during 8 min leg raising, the time to reach TAHO was estimated by extrapolation using an exponential function obtained by fitting the measured data of CSA-UA during 8 min leg raising.

## Results

The results of a power analysis with α = 0.05 and power = 0.8 show that to perform such a study to identify TAHO of OSA patients from the changes of the CSA-UA with fluid shift in 8 min, the required patient size is 18. Twenty-six candidates were recruited initially. One candidate withdrew after the polysomnography, and 2 patients quit during MRI due to claustrophobia. Data for 3 patients were excluded because of image blurring. The characteristics of the remaining 20 participants are given in Table 1.

**Table 1:** Baseline characteristics of 20 participants, including 19 men and 1 woman

Name of characteristics	min - max	mean ± SD
Age, y	29–63	48.05 ± 12.20
Weight, kg	67.0–120.6	82.13 ± 13.40
Height, m	1.65–1.86	1.74 ± 0.07
Body mass index, kg m^− 2^	22.60–39.70	27.2 ± 3.5
CSA-UA, cm^2^	0.26–1.54	0.80 ± 0.30
Neck circumference, cm	44.15–60.53	50.04 ± 4.64
Apnea-hypopnea index, events/h	16.5–67.0	41.36 ± 16.5

*CSA-UA* the narrowest cross-sectional area of the upper airway

Leg raising resulted in a volume expansion of the soft tissues in the neck region, which constricted UA. We estimated the changes in the tissue volume caused by fluid redistribution by measuring the changes in the cross-sectional area of the soft tissues from the images. Compared with the control, the cross-sectional area of the soft tissues in the narrowest retropalatal region increased 2.17 ± 1.53% after 8 min of leg raising due to additional fluid accumulation in that region. In the control stage, the CSA-UA of the patients was 0.80 ± 0.30 cm^2^, which was much smaller than 1.31 ± 0.68 cm^2^ measured in healthy subjects [17].

**Fig. 1:**
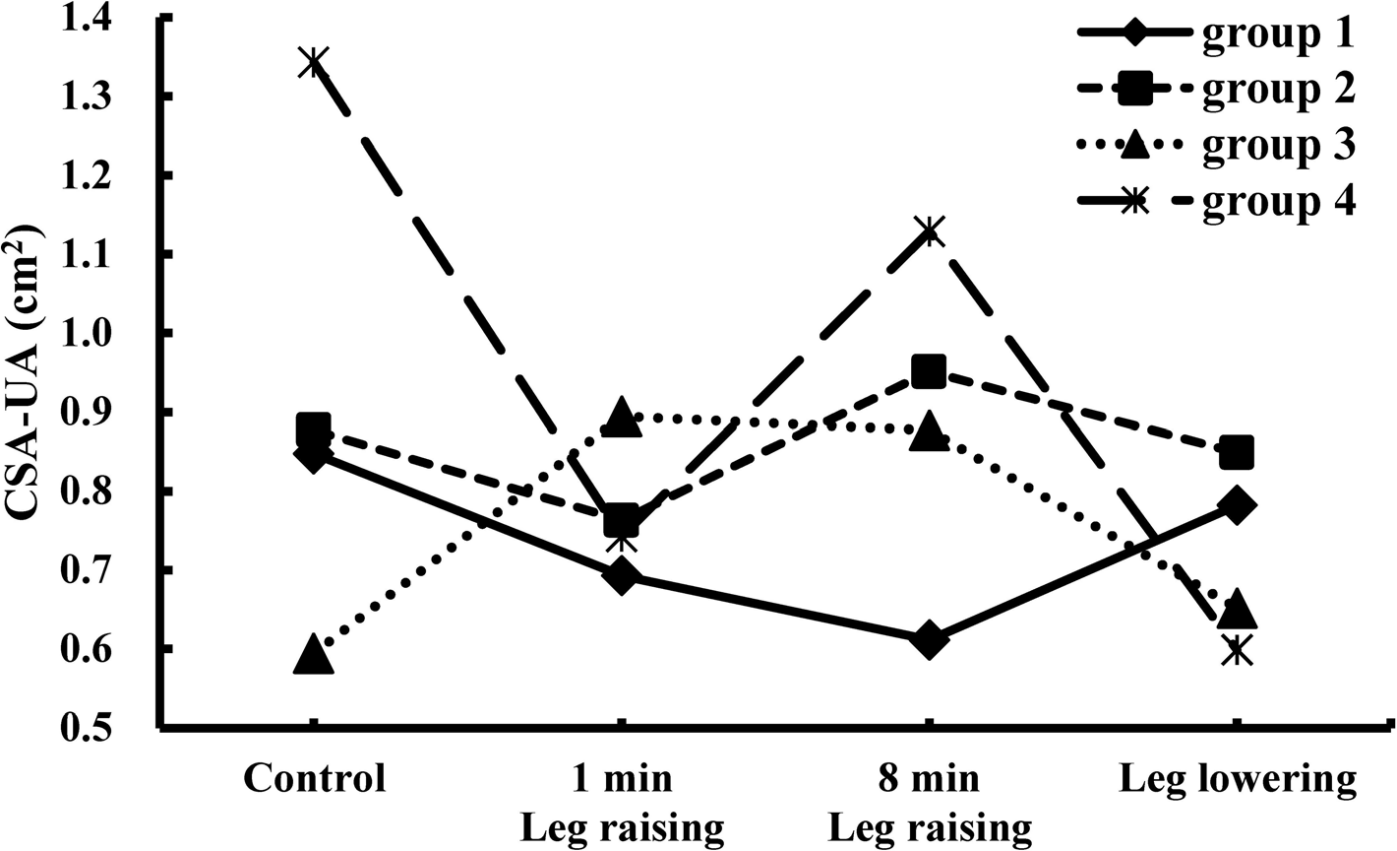
Four different types of changes in the cross-sectional area of the upper airway (CSA-UA) in OSA patients. The number of the patients in the four groups were 6, 4, 8, and 2 (30, 20, 40, and 10% of the total participants), respectively

**Fig. 2:**
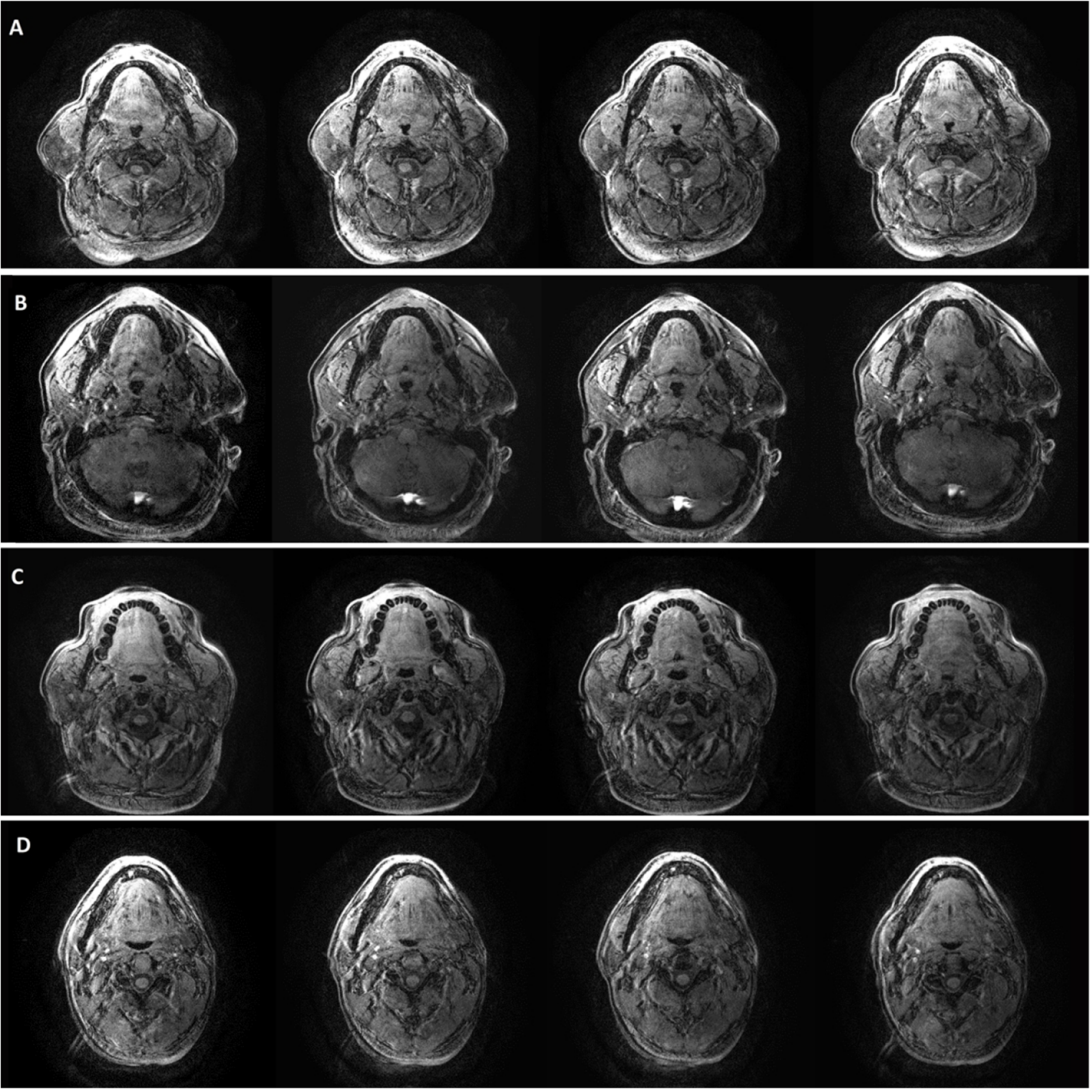
The cross-section of the upper airway at the same retropalatal level corresponding to the four scanning stages: the control, 1 min leg raising, 8 min leg raising, and leg lowering (from left to right). **a** to **d** were for groups 1 to 4 respectively

Contrasting the observations in healthy subjects, in which CSA-UA continuously decreased with the increase in fluid in the neck [17], we observed different types of changing patterns in CSA-UA in OSA patients as that we expected in our assumption. We divided the patients into four groups with 6, 4, 8, and 2 patients (30, 20, 40, and 10%), respectively, based on the characteristics of their CSA-UA variations during leg raising. Their CSA-UA data at each scanning stage are given in Table 2. As shown in Fig. 1, the first type, which shows a continuous decrease in CSA-UA during the fluid accumulation in the neck, is similar to that observed in healthy subjects [17]. The last three trends differed, which show a large increase in CSA-UA at a special time frame of the fluid redistribution procedure. Figure 2 shows the cross-section of UA at the same retropalatal level corresponding to the four scanning stages for each group. The variations of CSA-UA in groups 2 and 4 look similar in Fig. 1, but the airway size in the control was much larger than when lowering the legs in group 4. For each individual in groups 2, 3, and 4, we obtained TAHO by extracting the minimum value of CSA-UA curve. A mean TAHO 0.63 ± 0.21 cm^2^ was calculated using individual TAHO of 14 patients in these three groups.

**Table 2:** The measurements of the narrowest cross-sectional area (cm^2^) of the upper airway (CSA-UA) before and after elevating the legs

Group	Control	1 min leg raising	8 min leg raising	leg lowering
1 (*n* = 6)	0.85 ± 0.14	0.69 ± 0.16	0.61 ± 0.17	0.78 ± 0.19
2 (*n* = 4)	0.88 ± 0.33	0.76 ± 0.26	0.95 ± 0.30	0.85 ± 0.33
3 (*n* = 8)	0.59 ± 0.19	0.89 ± 0.32	0.88 ± 0.37	0.65 ± 0.23
4 (*n* = 2)	1.34 ± 0.27	0.74 ± 0.50	1.13 ± 0.12	0.60 ± 0.30

Data represent mean ± standard deviation. n: number of subjects

The results of correlation analyses show a positive correlation between TAHO and BMI (r = 0.681, *p* = 0.0007), and a negative correlation (r = − 0.513) between the reduction in CSA-UA and the increase in NC with the time of fluid shift, but the *p* value is a little bit larger than 0.05 (*p* = 0.051).

**Fig. 3:**
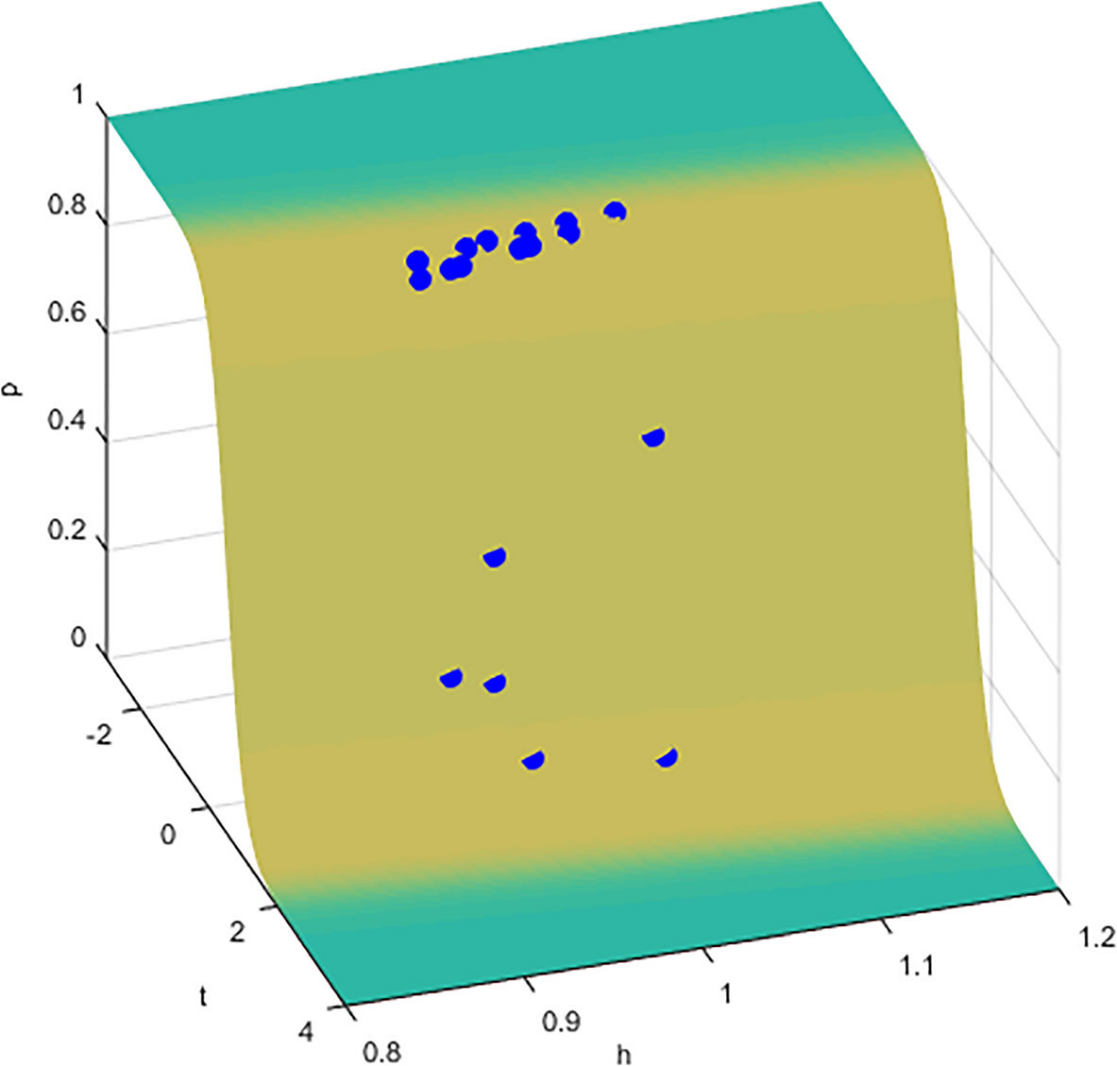
Predictions from a logistic analysis model on the probability of being a obstructive sleep apnea (OSA) patient for each of the 20 subjects. The sigmoid function for the probability of being a OSA patient *p* = 1/[1 + exp. (4.836 + 3.850 t-8.4 h)] is used to separate patients from normal subjects. Here, t is a dimensionless time variable defined as the time to reach the threshold divided by 10 min, and h is a dimensionless height variable defined as the height divided by 180 cm

A logistic function logit = − 4.836-3.850 t + 8.4 h or a sigmoid function for the probability of being a OSA patient *p* = 1/[1 + exp. (4.836 + 3.850 t-8.4 h)] was obtained to effectively separate OSA patients (labeled 1) from normal subjects (labeled 0). Here, t is a dimensionless time variable defined as the time to reach TAHO divided by 10 min, and h is a dimensionless height variable defined as the height divided by 180 cm. Figure 3 shows the prediction on the probability of being a OSA patient for each subject.

**Fig. 4:**
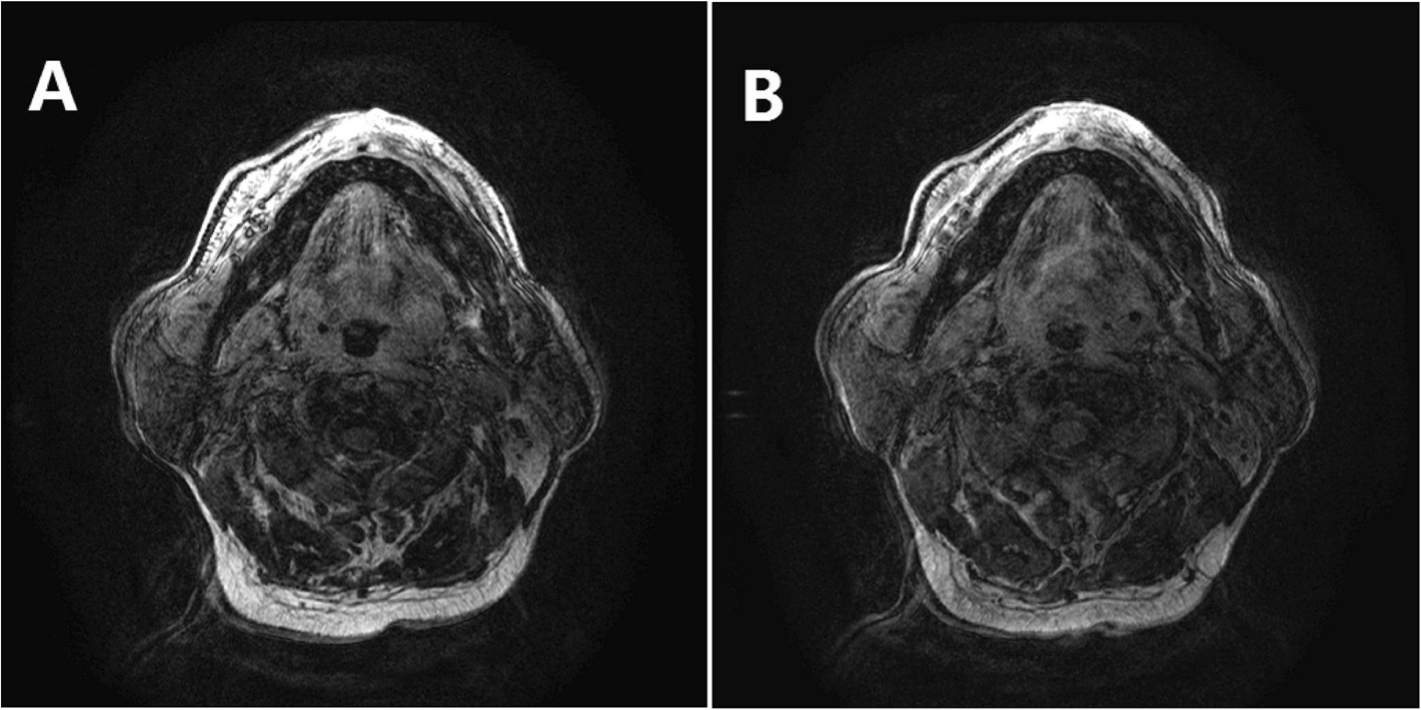
The shape change in the oropharynx cross-section of the upper airway after elevating the legs for 8 min. Compared to the control (**a**), the size of the airway in the anteroposterior direction decreased apparently, but the size in the transverse direction maintained almost unchanged after 8 min of leg raising (**b**)

**Fig. 5:**
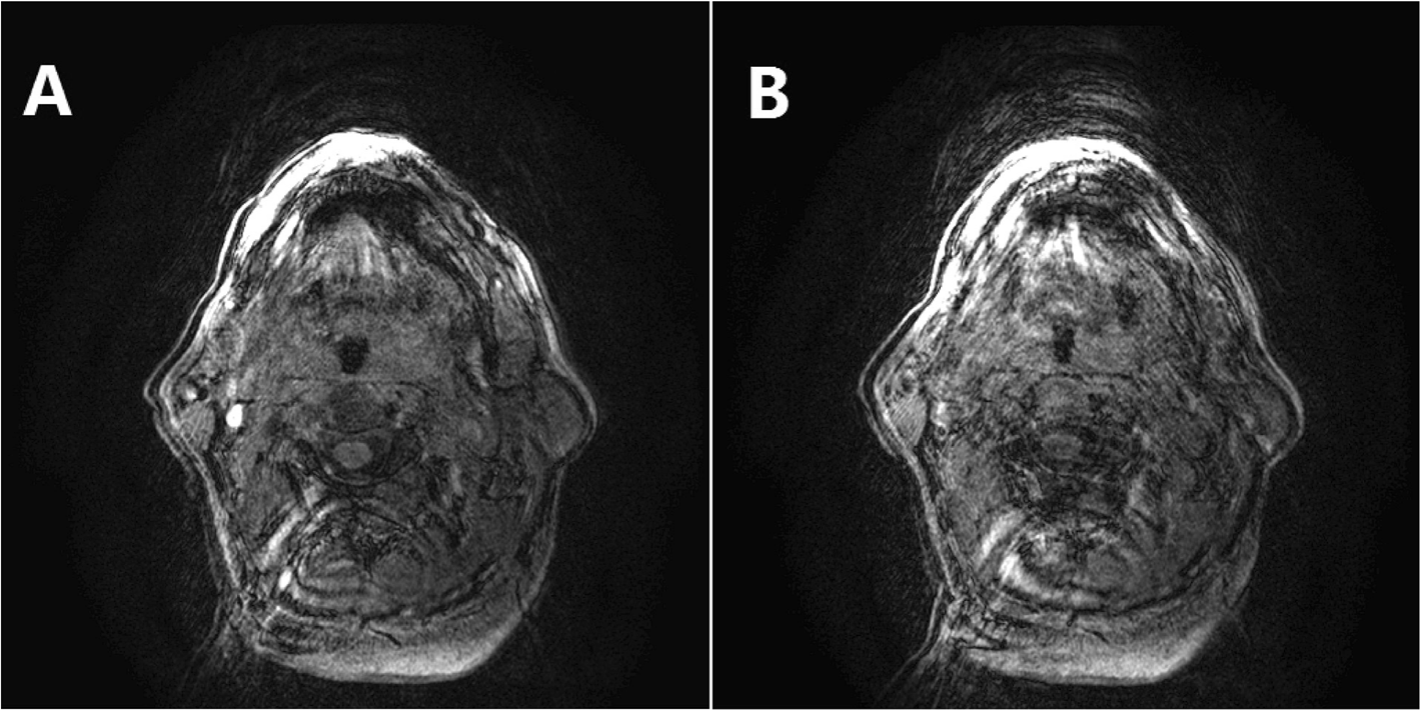
The shape change in the oropharynx cross-section of the upper airway after elevating the legs for 8 min. Compared to the reference (**a**), there was a significant size reduction in the lateral direction, while the change in the anteroposterior direction was small after 8 min of leg raising (**b**)

An important observation was the shape changes in UA cross-section caused by fluid redistribution. We observed the direction of the airway narrowing due to fluid accumulation, which is valuable in evaluating the behaviors of UA collapse. In addition to UA narrowing in both anteroposterior and lateral directions, Figs. 4 and 5 show two different tendencies of the shape change in the oropharynx cross-section of UA after 8 min leg raising. One was that the size of the airway in the anteroposterior direction decreased apparently, but the size in the transverse direction maintained almost unchanged compared to the control (Fig. 4). The other was that there was a significant size reduction in the lateral direction, while the change in the anteroposterior direction was small compared to the reference (Fig. 5). These results suggest that the patients with the first type of UA shape changes were more able to collapse their airway in the anteroposterior direction, but those with the second type of changes had a high probability of blocking their airway in the lateral direction during sleep.

Shape change could affect the flow resistance and the airway collapsibility significantly. If we classified the shape changes based on the ratio of the length to the short axis of UA cross-section, as that we did previously [8]: the ratio was a) basically unchanged, b) increased, and c) decreased. The percentage of the patients for cases a, b, and c was 50, 15, and 35%, respectively. Circular collapse fell into category a), accounting for 60% of this category. For the same cross-sectional area, an airway with a slender shape in the cross-section would have a larger resistance than that in a circular cross-section [8, 22].

## Discussion

In this study, we propose the important concept of CSA-UA threshold and develop an effective method to measure the parameter TAHO when awake, which can be used clinically as an indicator of hypopnea onset when asleep. We want to point out that although we call this parameter as a threshold for hypopnea, it is not limited to hypopnea only. Actually, once CSA-UA falls below TAHO during sleep, either hypopnea or apnea can occur if the airway continues to narrow. Using MRI, we successfully observed the increase in CSA-UA with fluid accumulation in the neck and identified their TAHO in 14 of the total 20 OSA patients. One may have noticed that although the 6 patients in group 1 did not show an increase in CSA-UA, as that behaved by 14 patients in groups 2, 3, and 4, during fluid redistributions, their CSA-UA at 8 min leg raising (Table 2 and Fig. 1) was quite close to the value of mean TAHO. This suggested that if the time of leg raising was slightly longer than 8 min, one may see CSA-UA increase in more patients. From this threshold concept, we can well explain all observations in Fig. 1. For group 1, CSA-UA is large enough to maintain normal breathing without a requirement of additional muscle activity during the leg raising. Although decreasing, CSA-UA still maintains to be above TAHO, with the increase in the amount of fluid when awake, as observed in normal subjects [17]. Once CSA-UA reaches a critical value TAHO, as happened between 1 min and 8 min leg raising in group 2, and between the control and 1 min leg raising in group 3, the narrowing airway cannot fulfill the requirement for normal breathing, and the pharyngeal dilator muscle activations are enhanced to increase UA size. For patients in group 4, although CSA-UA is above TAHO when standing, an increase in the amount of fluid in the neck caused by lying down decreases airway size to below TAHO, triggering strong muscle activation to enlarge the airway. When lowering the legs, because they cannot recover immediately to their control status, the airway size can be slightly larger than TAHO.

A positive correlation between TAHO and BMI obtained in this study suggests that weight gain may increase TAHO, and therefore, increase the risk of hypopnea and even OSA because a large TAHO value means that a large airway cross section is required for normal breathing. A negative correlation between the reduction in CSA-UA and the increase in NC with the time of fluid shift, although the *p* value is a little bit larger than 0.05, presents a tendency that a larger increase in NC corresponds to a smaller reduction in the airway size, and a smaller increase in NC corresponds to a lager reduction in the airway caliber. This is not surprising, and it is consistent with our previous studies [17]. MRI Studies have revealed that the tissue volume increase caused by rostral fluid shift can have different consequences: increasing NC, narrowing the upper airway, or both [17]. This is because the neck skin limits the expansion of the tissue volume, and therefore modulates CSA-UA and NC [18]. Because the elastic modulus of the neck skin is different among individuals [18], for those who have soft neck skin with a lower elastic modulus, the fluid accumulated in the neck will lead mainly to an outward expansion of the neck, and thus, its impact on UA size is relatively small. For those who have hard neck skin with a larger elastic modulus, it is difficult to expand NC when fluid accumulates in the neck, and as a result, the accumulated fluid will mainly force the soft tissue to move toward UA and result in the airway narrowing. The results observed in this study suggest that the patients with small NC increase will have more reduction in CSA-UA with the increase in the volume of soft tissues surrounding the upper airway, and therefore it is easier for the airway to be affected by weight changes and rostral fluid redistributions.

The potential clinical significance of TAHO is clear. TAHO can be conveniently obtained while awake by measuring the minimum value of CSA-UA versus fluid accumulation curve for OSA patients. This is because UA of patients is narrow. When the fluid in the legs shifts into the neck region under waking condition, the volume expansion of the soft tissues, caused by the increased fluid amount, will compress the airway so that CSA-UA can easily fall below the threshold, triggering dilator muscle activations. Compared to patients, when a similar amount of fluid accumulates in the head and neck region of normal subjects, their airway size does not fall below the threshold [17]. Therefore, this is a convenient daytime evaluation method, as shown in the logistic regression model, to predict the possibility of incurring hypopnea or even OSA during sleep for individuals. By measuring the time reaching TAHO and using the probability function, one can identify OSA patients under waking condition. TAHO is parameter measured in the supine position, which is meaningful for patients with and without positional sleep apnea. Patients with positional OSA generally have more OSA symptoms when they sleep with a supine position than with a lateral position. A major reason is that the uvula will lean to one side due to gravity when people sleep with the lateral posture, and therefore increase the cross-sectional area of the airway at that particular location, which is generally the position with the narrowest cross-section of UA. If CSA-UA is above TAHO due to a lateral position, normal breathing can be maintained, and hypopnea or OSA will not occur. However, if the cross-sectional area still falls below TAHO when sleeping with a lateral position, there is a risk of hypopnea and even OSA.

For a potential OSA patient, a further diagnosis may be done in the following way: After measuring the personal TAHO using the method developed in this study, one can reduce the ability of genioglossal muscle activity of the subject to a level similar to that during sleep in a traditional way, for example via local anaesthesia of the tongue muscle. One can then measure CSA-UA under such a condition and compare it with TAHO to determine whether OSA will happen during sleep.

For a diagnosed OSA patient, TAHO can be used to optimize the design of personalized non-CPAP treatment by predicting the efficiency of a treatment design in advance, and therefore increase the probability of success. To predict whether a treatment is effective, the key is to determine whether CSA-UA of the individual can fall below its threshold during sleep after such a treatment. Currently, a potential method for predicting therapeutic outcomes is to perform the numerical simulation using computer models. Combining medical imaging with finite element analysis models, one can simulate the process of upper airway collapse and obstruction corresponding to the personalized treatment design [23–26]. A computational model with realistic head and neck anatomy can be built for a patient, and then a treatment plan can be designed to enlarge the upper airway by physically expanding the facial skeletal framework or excising some tissues, such as uvulopalatopharyngoplasty surgery or maxillomandibular advancement. By numerically simulating the deformation and collapse of the upper airway under a significantly reduced genioglossal muscle activity, which can be simulated using the mechanical model of the tongue muscle [24], one can evaluate whether CSA-UA can be maintained above TAHO after such a treatment, and therefore, judge the effectiveness of this particular treatment design. By comparing the simulation results of various treatment designs, the most effective personalized treatment plan for this patient can be determined, and all designs that cannot work effectively can be eliminated. This prediction procedure can greatly reduce the uncertainty of treatment and improve the success rate.

Using MRI, we can obtain not only the value of CSA-UA, but also the cross-sectional shape of UA. By observing the shape changes caused by fluid redistribution, we can identify different types of UA collapse among patients. As shown in this study, someone had the major UA collapse in the anteroposterior direction, someone had the major collapse in the lateral, and someone had circular collapse. This individual information, obtained under waking condition, is particularly important when designing a personalized surgery for a patient.

A limitation of this study is that we could not measure genioglossal muscle activation simultaneously when performing MRI. Therefore, we could not provide actual electromyogram data corresponding to CSA-UA increase at the current stage, and more tests are needed in future studies. Another limitation is that we have only four scanning stages, and an airway enlargement may happen between two scanning stages. Therefore, the real TAHO may be lower than that obtained in this study. Our value is actually an upper limit of TAHO. For more accurate measurement on TAHO, one needs to shorten the time interval between two scanning stages.

## Conclusions

The important findings in this study prove our hypothesis on the threshold of the narrowest cross-sectional area of UA. The threshold measured while awake for normal breathing can be used clinically as the indicator of hypopnea onset during sleep. Both shape and size changes in UA cross-section can affect airway resistance significantly. Shape change in UA cross-section can provide key clinical information on UA collapse patterns for individuals. The concept of CSA-UA threshold for hypopnea onset during sleep, the method to measure the parameter TAHO when awake and to identify OSA patients based on TAHO, and UA collapse tendency judged by the cross-sectional shape change of UA will lead to new insights into the development of new methods for OSA diagnosis and treatments.

## Data Availability

The datasets used and/or analysed during the current study are available from the corresponding author on reasonable request.

## Abbreviations

AHI: the apnea-hypopnea index
BMI: the body mass index
CSA-UA: the narrowest cross-sectional area of the upper airway
NC: the neck circumference
OSA: Obstructive sleep apnea
TAHO: The threshold of the area for hypopnea onset
UA: the upper airway
